# IFN‐γ Driven Hepatic Injury Exacerbates Mortality in NK/T‐Cell Lymphoma‐Associated Hemophagocytic Lymphohistiocytosis

**DOI:** 10.1002/cam4.71606

**Published:** 2026-02-15

**Authors:** Yehua Yu, Liyuan Ma, Haifang Hang, Yuyang Pang, Wei Lu, Jiajia Liu, Hui Zhou, Jun Shi

**Affiliations:** ^1^ Department of Hematology Shanghai Jiao Tong University School of Medicine Affiliated Ninth People's Hospital Shanghai China

**Keywords:** emapalumab, hemophagocytic lymphohistiocytosis, hepatic impairment, IFN‐γ, NK/T‐cell lymphoma

## Abstract

**Background:**

Hepatic involvement is a life‐threatening complication in natural killer/T‐cell lymphoma with hemophagocytic lymphohistiocytosis (NK/T‐HLH). Nevertheless, the prognostic implications of hepatic dysfunction and its underlying related factors are not fully elucidated.

**Methods:**

We retrospectively analyzed the clinical and laboratory data of 53 NK/T patients, comprising 35 cases without HLH and 18 with HLH. All NK/T‐HLH patients exhibited hepatic injury, characterized by significantly elevated hepatic enzyme levels and bilirubin compared to those without HLH (all *p* < 0.001). Glutamyltransferase (GGT), aspartate aminotransferase (AST), and direct bilirubin (DBIL) individually predicted mortality with AUC > 0.8 in NKTCL patients, while their composite GSD index enhanced predictive accuracy (AUC = 0.87).

**Results:**

Notably, NK/T‐HLH patients meeting the GSD index criteria had markedly reduced overall survival (OS) compared to non‐fulfillers (median OS: 2 vs 21 months; *p* = 0.003). Patients with hepatic involvement exhibited significantly higher levels of serum interferon‐γ(IFN‐γ) and interleukin‐10 (IL‐10). While, only IFN‐γ concentrations showed a strong positive correlation with the elevated levels of GGT, AST, and DBIL. To further validate the clinical relevance of these findings, we present two representative cases of NK/T‐HLH with severe hepatic injury. Both patients achieved rapid liver function recovery following a targeted regimen combining chemotherapy and emapalumab, a human anti‐IFN‐γ monoclonal antibody approved for primary HLH.

**Conclusion:**

The GSD index emerges as a robust prognostic tool for NK/T‐HLH patients with hepatic dysfunction, reflecting underlying IFN‐γ‐mediated immunopathology. Early intervention with anti‐IFN‐γ monoclonal antibody may improve clinical outcomes in this high‐risk subgroup.

## 1 Introduction

Natural killer/T‐cell lymphoma (NKTCL) is an aggressive and heterogeneous subtype of non‐Hodgkin lymphoma. Approximately 11.4% of patients with NKTCL will develop hemophagocytic lymphohistiocytosis (HLH) [1], a highly life‐threatening clinical hyperinflammatory syndrome. Standard presenting features of HLH include fever, splenomegaly, cytopenia, coagulopathy, and hemophagocytosis in bone marrow (BM) or other organs. HLH is a leading cause of death among patients who are newly diagnosed or have advanced NKTCL [2]. The 2‐year overall survival (OS) rate for natural killer/T‐cell lymphoma with hemophagocytic lymphohistiocytosis (NK/T‐HLH) ranges from 4.8% to 16% [3, 4].

More than 70% of patients with HLH exhibit varying degrees of hepatic involvement [5]. HLH patients who experience acute liver failure (ALF) have an inferior prognosis, with a mortality rate as high as 92.9% [6]. Abnormal levels of liver enzymes, bilirubin, or albumin (ALB) serve as indicators of hepatic impairment. Some studies suggest elevated alanine aminotransferase (ALT) or bilirubin levels do not provide valuable prognostic information for HLH [7]. Conversely, other research revealed that direct bilirubin (DBIL) and aspartate aminotransferase (AST) were associated with poor outcomes in adult HLH patients [8, 9]. Wei Sang et al. confirmed that albumin and ALT were independent prognostic factors of adult HLH [10]. Therefore, it remains unclear which parameters of hepatic impairment could serve as precise predictive indices for the prognosis of NK/T‐HLH.

Ruxolitinib and the HLH‐94 or DEP regimen (which includes liposomal doxorubicin, etoposide, and high‐dose methylprednisolone) are currently considered therapeutic options for patients with NKTCL who have HLH [11, 12, 13]. However, there is limited clinical experience in treating HLH in the context of liver failure. Chemotherapy can induce hepatotoxicity, which makes finding alternative treatments necessary for patients with severe hepatic impairment. The combination of glucocorticoids, etoposides, fludarabine, and gamma globulin did not yield satisfactory outcomes in HLH patients with liver failure, as evidenced by a survival rate of only 45.45% in a retrospective study [14]. Cytokine storm, an immune dysregulation characterized by constitutional symptoms, systemic inflammation, and multiorgan dysfunction, can lead to multiple organ failure if not adequately addressed [15]. This phenomenon is widely recognized as a direct contributor to the development of HLH. It can cause damage across various organs [16]. However, it remains unclear whether the hepatic impairment seen in NK/T‐HLH patients results from the cytokine storm and which specific cytokines are most closely associated with this condition.

In this study, we evaluated the impact of hepatic involvement on the prognosis of NK/T‐HLH patients and analyzed factors related to hepatic impairment in NKTCL patients. Additionally, we shared the experience of two patients with NK/T‐HLH and ALF who were successfully treated with emapalumab. This is the first international report on using interferon‐γ (IFN‐γ) monoclonal antibody for treating NKTCL patients with HLH.

## 2 Patients and Methods

Patients diagnosed with extranodal NKTCL at the Ninth People's Hospital affiliated with Shanghai Jiao Tong University from 01/07/2018 to 30/06/2024 were retrospectively reviewed. The inclusion criteria for this study were as follows: (1) Adult patients aged between 18 and 80 years. (2) Patients who had complete treatment history and essential clinical data. The exclusion criteria were: (1) History of malignant tumors other than NKTCL unless the patient has been cured for over 3 years. (2) Patients developing liver injury only after hepatitis virus infection. Patients meeting the screening criteria were recruited to participate in the study. Due to the unavailability of the NK cell activity assay at our hospital, we classified patients who met at least 5 out of the 7 diagnostic criteria from the HLH‐2004 guidelines as having NK/T‐HLH [3]. Those with fewer than five criteria were categorized as having NK/T‐NHLH. Clinical outcomes were monitored until the end of December 2024.

### 2.1 Data Collection

The medical records of all patients were reviewed for demographic characteristics and clinical and laboratory findings. Demographic and clinical characteristics included age at diagnosis, gender, presence or absence of fever, splenomegaly, and hepatomegaly. Laboratory findings included complete blood count, liver indices, lactate dehydrogenase (LDH), coagulation function, immune cell subsets, and serum cytokine levels. Positron emission tomography/computed tomography (PET‐CT), BM smears and biopsy, and lumbar puncture to examine the cerebrospinal fluid. Liver indices included GGT, ALT, AST, DBIL, indirect bilirubin (IBIL), and ALB. Immune cell subsets included CD3+ T cells, CD3+CD4+ T cells, CD3+CD8+ T cells, CD19+ B cells, and CD16+CD56+ NK/T cells. Cytokines included interleukin‐2 (IL‐2), IL‐4, IL‐5, IL‐6, IL‐8, IL‐10, IL‐17, IL‐12P70, IL‐1β, IFN‐α, tumor necrosis factor‐α (TNF‐α), and IFN‐γ. Laboratory parameters for NK/T‐NHLH patients were recorded on the day of hospital admission. For patients who developed HLH during hospitalization, parameters were captured within 7 days preceding the HLH diagnosis. The lymphoma stage was assessed using the Ann Arbor Staging System. Risk stratification was determined according to the nomogram‐revised risk index (NRI) scores [17]. The treatment response of HLH was evaluated according to the response criteria of Marsh et al. [18]. Hepatic involvement was defined by meeting ≥ 1 of the following ACG 2021 guideline criteria: elevated GGT (> 61 U/L in men, > 36 U/L in women), elevated ALT (> 33 U/L)/AST (> 35 U/L), hyperbilirubinemia (total bilirubin > 1.2 mg/dL), hypoalbuminemia (albumin < 3.5 g/dL) [19]. The primary outcome variable was OS. OS was measured from the diagnosis date to the date of all‐cause death or last follow‐up.

### 2.2 Statistical Analysis

Statistical analyses were performed using GraphPad Prism, version 9.3.1 (San Diego, CA). The Mann–Whitney U test was employed to compare continuous variables, while the Fisher's exact test or Chi‐square test was used to compare categorical parameters, as applicable. The area under the curve (AUC), sensitivity, specificity, and optimal cutoff levels were calculated using receiver‐operating curves (ROC) for mortality prediction. An AUC > 0.8 reflects a test with good discriminatory ability. We calculated the optimal cutoff point for each parameter chosen as the point with the highest Youden index (sensitivity + specificity−1). Binary logistic regression was performed using the highest probability markers to identify mortality at 180 days. The combined ROC curve was produced using the predicted probability as the test variable. Internal validation and sensitivity analysis were performed using bootstrap resampling. Variables with *p* < 0.1 in univariate analysis were entered into a multivariable Cox proportional hazards model to identify independent prognostic factors. Survival time was estimated using the Kaplan–Meier method. The correlation analysis was assessed using Pearson's *r*‐test. Statistical significance was defined by *p < 0.05*.

## 3 Results

### 3.1 Patient Characteristics and Survival Analysis

**FIGURE 1:**
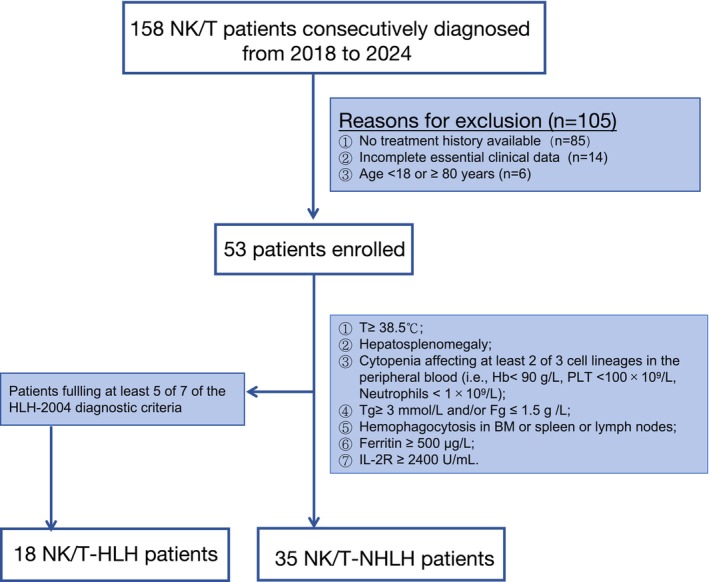
Flowchart of NK/T cases included in the study.

A total of 53 patients were enrolled in the analysis. Figure 1 presents the flowchart illustrating this study's exclusion and group assignment processes. The patient population consisted of 36 males and 17 females, with a median age of 49 (ranging from 29 to 79). Eighteen patients were classified as NK/T‐HLH, while the remaining 35 were classified as NK/T‐NHLH. Among NK/T‐HLH patients, 13 had HLH at the initial diagnosis, while five developed HLH as the lymphoma progressed.

The clinical characteristics are summarized in Table 1. No differences were found in gender or age distribution between HLH and NHLH groups. Fifty percent of NK/T‐HLH patients presented as non‐nasal type, and 83.3% were classified as Ann Arbor stage III/IV, significantly higher than those with NK/T‐NHLH (*p* = 0.032, *p* = 0.0003, respectively). HLH was more likely to appear in NKTCL patients in the high‐risk group (*p* = 0.005). The frequency of BM and CNS involvement patients was higher in NK/T‐HLH than in NK/T‐NHLH (38.9% vs. 5.7%, *p* = 0.005 and 27.8% vs. 2.9%, *p* = 0.014). Notably, hepatic involvement was observed in 62.3% of NKTCL patients, where all 18 NK/T‐HLH patients had varying degrees of hepatic impairment.

**TABLE 1:** Clinical characteristics in patients with NK/T‐HLH and NK/T‐NHLH.

Characteristics	NK/T‐HLH	NK/T‐NHLH	*p*
Number of patients, *n*	18	35	
Gender, *n* (%)			
Male	12 (66.7)	24 (68.6)	> 0.9999
Female	6 (33.3)	11 (31.4)	
Age (years), Median (range)	49 (29–79)	54 (19–79)	0.812
Primary lesion location, *n* (%)			
Nasal	9 (50)	28 (80)	**0.032**
Non‐nasal	9 (50)	7 (20)	
Prognosis group, *n* (%)			
Low‐risk	1 (6)	8 (22.8)	**0.016**
Intermediate‐risk	1 (6)	10 (28.6)	
High‐risk	16 (88)	17 (48.6)	
Ann Arbor stage, *n* (%)			
I–II	3 (16.7)	25 (71.4)	**0.0003**
III–IV	15 (83.3)	10 (28.6)	
BM involvement, *n* (%)	7 (38.9)	2 (5.7)	**0.005**
CNS involvement, *n* (%)	5 (27.8)	1 (2.9)	**0.014**
Hepatic involvement, *n* (%)	18 (100)	15 (42.9)	**< 0.0001**
Fever, *n* (%)	18 (100)	13 (37.1)	**< 0.0001**
Hepatosplenomegaly, *n* (%)	9 (50)	8 (22.9)	0.064
Hemophagocytosis in BM, *n* (%)	14 (77.8)	2 (5.7)	**< 0.0001**
Epstein–Barr virus (EBV) in blood			
Negative	3 (20)	11 (36.7)	0.321
Positive	12 (80)	19 (63.3)	

### 3.2 A Hepatic Impairment Index Comprising GGT, AST, and DBIL Can Predict Mortality in NKTCL Patients

**FIGURE 2:**
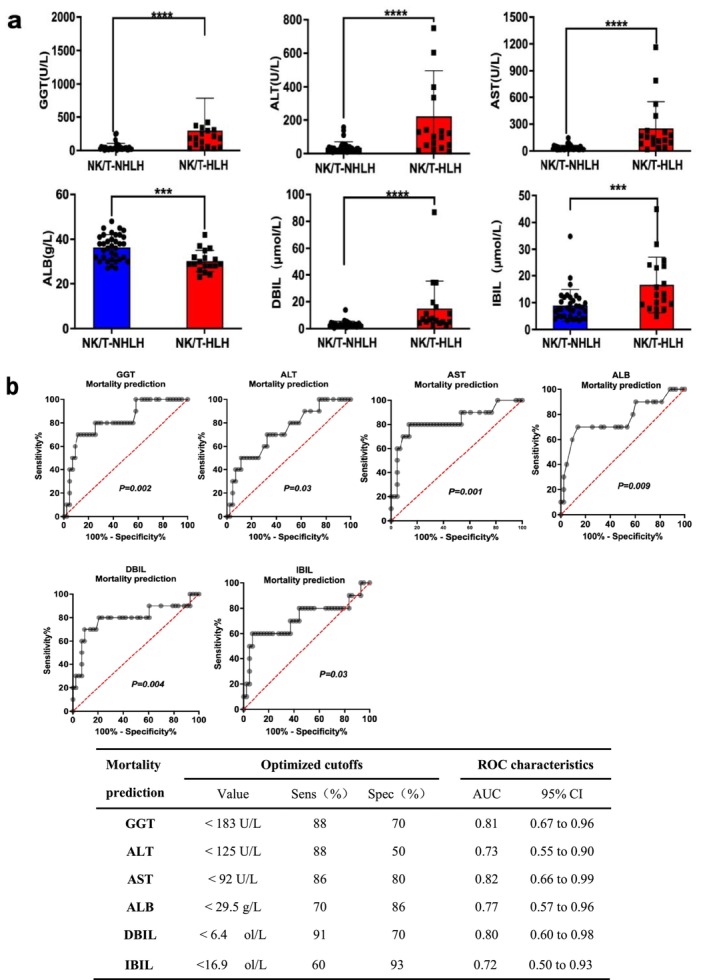
Hepatic parameters can act to predict mortality in patients with NKTCL. (a) Difference of hepatic parameters among NKTCL patients with or without HLH; ***p < 0.001, ****p < 0.0001. (b) ROC for the prediction of mortality at 6 months in NKTCL patients; The dotted line indicates the pertinent point on the curve identified as the best balance between sensitivity (Sens) and specificity (Spec) (with the highest Youden index). The table shows the sensitivity and specificity of each hepatic marker for mortality.

We assessed the distribution of hepatic parameters among NKTCL patients with or without HLH. Notably, NK/T‐HLH patients exhibited markedly elevated GGT, ALT, AST, DBIL, and IBIL compared to NK/T‐NHLH patients. Moreover, the ALB levels in NK/T‐HLH patients were lower than those in the NK/T‐NHLH group (Figure 2a). To identify critical indicators of hepatic impairment predictive of prognosis in patients with NKTCL, we performed ROC analyses of hepatic parameters to predict mortality in the cohort. Notably, GGT, AST, and DBIL showed sufficient discriminatory power for predicting mortality over the subsequent 6 months, with the AUCs exceeding 0.8 (Figure 2b).

**FIGURE 3:**
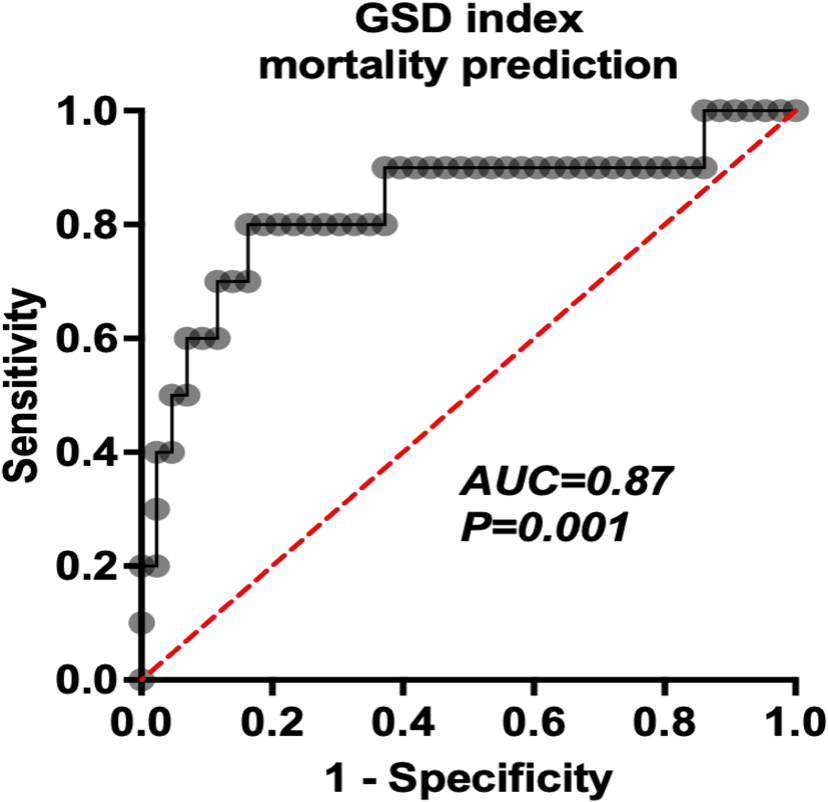
The GSD index can improve mortality prediction in patients with NKTCL.

To improve the significance of hepatic impairment in mortality prediction, we combined these three markers of GGT, AST, and DBIL as the GSD index and derived the cutoffs of these three ROCs as 183 U/L for GGT, 92 U/L for AST, and 6.4 μmol/L for DBIL. The value of predicting mortality at 6 months was assessed with binary logistic regression. Indeed, this resulted in an improved mortality prediction (AUC = 0.87, sensitivity, 82%; specificity, 81%) (Figure 3), which is superior to any of GGT, AST, and DBIL considered alone. Internal validation via 1000 bootstrap resamples demonstrated stable predictive performance of the GSD index (mean AUC = 0.76, *p* = 0.000; ROC in Figure S1). Sensitivity analyses across varying resample sizes (1000–10,000) showed consistent confidence intervals and significance levels, confirming result stability (Table S1).

### 3.3 The GSD Index Can Be an Important Prognostic Factor in NKTCL Patients With HLH

**FIGURE 4:**
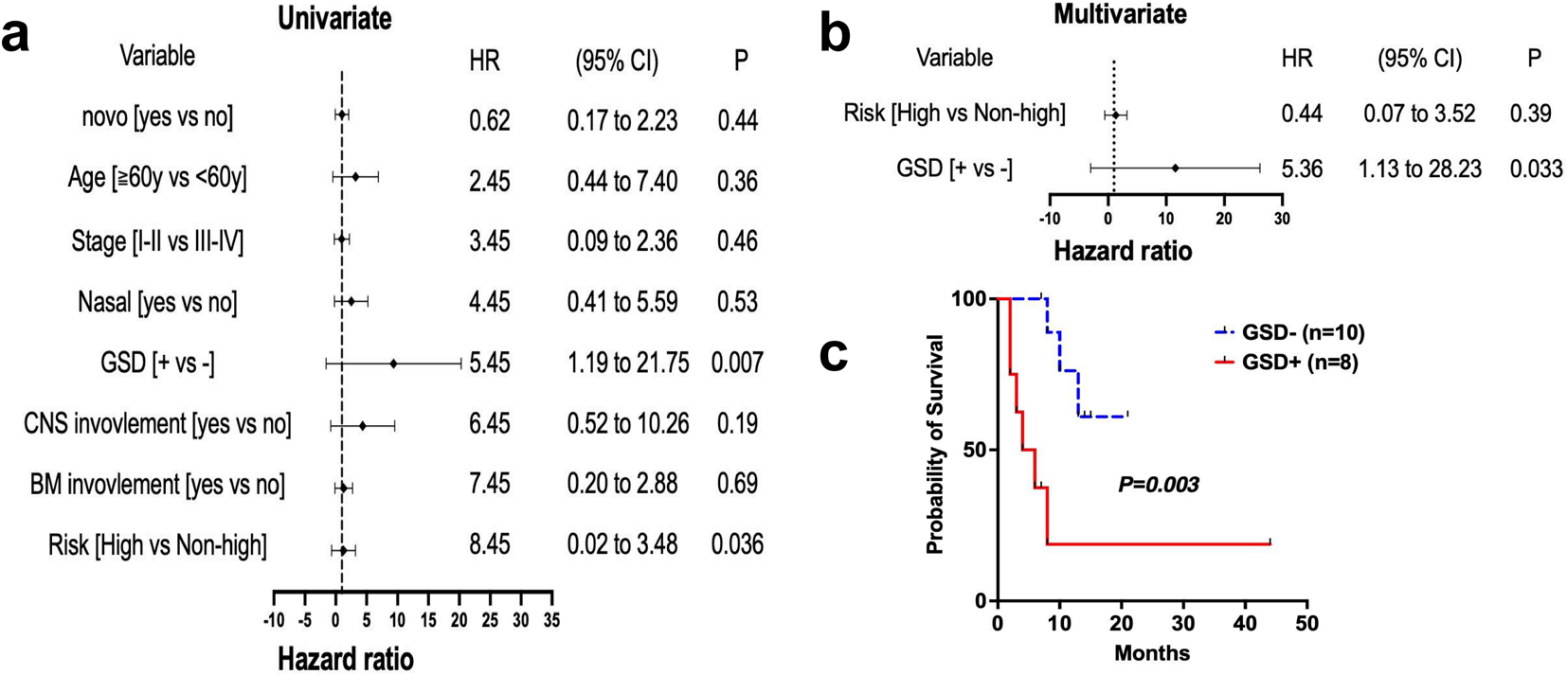
The GSD index can be an important prognostic factor in patients with NK/T‐HLH. (a) Univariate analysis for NK/T‐HLH related risk factors. (b) Multivariate analysis for NK/T‐HLH related risk factors. (c) Analyses of OS in NK/T‐HLH patients with positive versus negative GSD index.

We categorize patients into GSD index positive and GSD index negative groups based on predefined cutoff values for GGT, AST, and DBIL. Univariate analysis was performed to evaluate the prognostic significance of GSD index, disease stage, risk stratification, and other factors that are recognized as prognostic indicators in HLH patients. The results indicated that a positive GSD index and high‐risk stratification were significantly associated with poor survival outcomes (*p* = 0.007 and 0.036, respectively) (Figure 4a). Furthermore, Cox multivariate analysis confirmed that a positive GSD index was an independent predictor of outcome in patients with NKT‐HLH (*p* = 0.033) (Figure 4b).

With a median follow‐up of 12 months (ranging from 2 to 57 months) for all patients, the 1‐year OS was 76.3%; for NK/T‐HLH patients, the median follow‐up was 8 months (ranging from 2 to 44 months) with a 1‐year OS of 42.9%. Among the 53 patients included in the analysis, 39 patients (73.6%) were censored at last contact (14 lost to follow‐up; 25 alive at study cutoff). Baseline characteristics showed no significant differences between study completers and lost‐to‐follow‐up cases (Table S2; all *p* > 0.05). Fourteen patients died since diagnosis, including 10 patients with NK/T‐HLH and four patients with NK/T‐NHLH; NK/T‐HLH patients who met the GSD index criteria demonstrated significantly worse OS outcomes compared to those who did not. The median survival times for these two groups were 2 and 21 months, respectively (Figure 4c).

### 3.4 Cytokines Correlating With Hepatic Injury in NKTCL Patients

**FIGURE 5:**
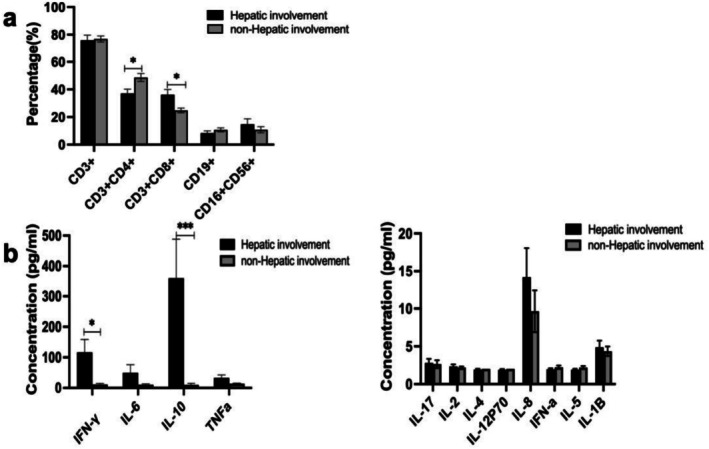
Percentage of immune cell subsets (a) and cytokine profiles (b) in NKTCL patients with hepatic involvement or not; *p < 0.05, ***p < 0.001.

To investigate the potential association between hepatic involvement and cytokine profiles in NKTCL patients, we compared immune cell subsets and cytokine levels between NKTCL patients with and without hepatic involvement. Our results revealed that NKTCL patients with hepatic involvement exhibited a significantly lower proportion of CD3+CD4+ T cells and a higher proportion of CD3+CD8+ T cells than those without hepatic involvement. In contrast, the proportions of CD16+CD56+ NK/T cells were comparable between the two groups. Within the cohort, cytokine levels were unavailable for seven patients. Baseline characteristics showed no significant differences between cases with and without cytokine data (Table S3, all *p* > 0.05). In the analyzed patients, levels of IFN‐γ and IL‐10 were significantly elevated in NKTCL patients with hepatic involvement compared to those without. The median (range) of IFN‐γ and IL‐10 in NKTCL patients with hepatic involvement were 32.87 (1.1–743.56) pg/mL and 47.96 (2–2350.1) pg/mL, respectively (Figure 5).

**FIGURE 6:**
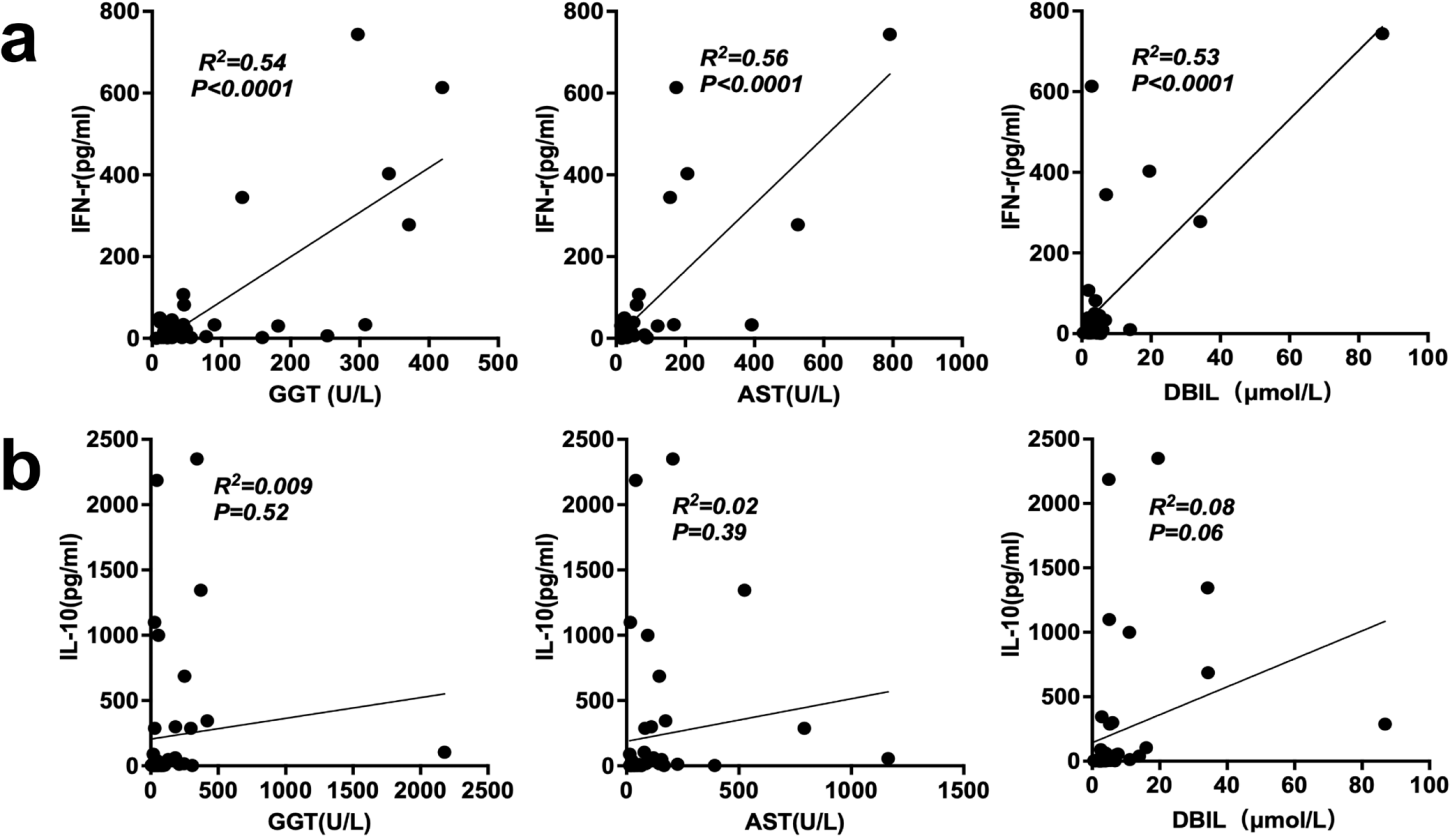
Linear correlation analysis of GSD parameters with IFN‐γ (a) and IL‐10 (b) in NKTCL patients.

Subsequently, we performed a correlation analysis to examine the relationship between GSD parameters and the levels of IFN‐γ and IL‐10. Intriguingly, our analysis revealed a strong positive correlation between IFN‐γ and elevated GGT, AST, and DBIL levels, as illustrated in Figure 6a. In contrast, IL‐10 showed no significant correlation with any of the GSD parameters, as depicted in Figure 6b.

### 3.5 Emapalumab Administration to HLH With Severe Liver Impairment in NKTCL Patients

**FIGURE 7:**
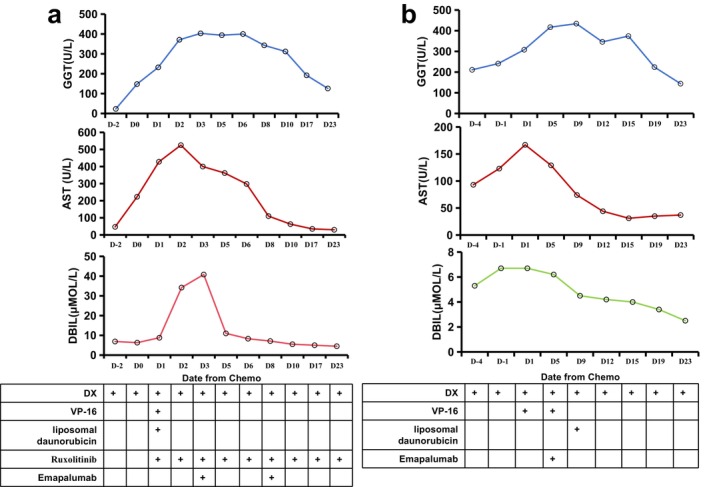
Changes in liver function after emapalumab combined with chemotherapy for two NK/T‐HLH patients. (a) Serial GGT, AST, and DBIL of No. 1 patient in Table S2 during treatment for HLH. (b) Serial GGT, AST, and DBIL of No. 2 patient in Table S2 during treatment for HLH.

The treatment and response outcomes of 18 NK/T‐HLH patients were listed in Table S2. Seventeen patients were given glucocorticoid (dexamethasone or prednisone); patients also received etoposide (VP‐16, *n* = 14), ruxolitinib (*n* = 2), and emapalumab (*n* = 3). Eight (44.4%) fulfilled the GSD index criteria of all patients presenting with hepatic involvement. Following intensive therapy, objective responses were observed in 10/18 (55.6%) cases, with five achieving complete response (CR) and five attaining partial response (PR) according to the HLH response criteria. Patients who achieved a response were promptly referred for treatment of the lymphoma. One patient died from disease progression before treatment, and nine died from inadequate disease control. Two patients with NK/T‐HLH experienced severe liver impairment. To avoid further damage to the liver from glucocorticoid and cytotoxic chemotherapy drugs, emapalumab was used after DX and VP‐16. The recommended starting dose of emapalumab is 1 mg/kg via intravenous infusion twice weekly. In this study, a fixed dose of 50 mg was administered per infusion due to cost considerations. One patient received a single 50 mg dose, and the other received two 50 mg doses. Treatment was discontinued upon documentation of significant hepatic improvement. All administrations were supplemented with prophylactic antimicrobial therapy (acyclovir, fluconazole, sulfamethoxazole). Emapalumab infusions were well tolerated, with no observed infections, infusion‐related reactions, or other adverse events in either patient during therapy. The liver function of these two patients improved rapidly after the administration of emapalumab (Figure 7). One of the two patients is currently alive and being treated for lymphoma; the other one died from severe pancreatitis.

## 4 Discussion

The most fatal complication associated with NKTCL is HLH. In this study, 71.4% of deaths occurred in NKTCL patients with HLH, which is similar to what many other studies have documented [1]. Hepatic involvement is a common clinical feature of HLH [20], but its prognostic impact on NK/T‐HLH patients remains unclear. Here, we devised a hepatic impairment index for predicting mortality in NKTCL patients for the first time. Additionally, we analyzed the potentially correlated cytokines of hepatic impairment and provided a therapeutic strategy to improve the prognosis of NK/T‐HLH patients with severe hepatic injury.

Previously published data showed 7.1%–57.1% of NKTCL patients presented with HLH at lymphoma diagnosis or upon progression [1, 21]; in our current study, the incidence was 34%. All the NK/T‐HLH patients presented with heavy tumor burden, advanced stage, and were more likely to be diagnosed with organ involvement. Notably, 100% of NK/T‐HLH patients have hepatic impairment. This high incidence may be attributed to our diagnostic criteria, which classified NKTCL patients meeting at least 5 of the 7 HLH‐2004 criteria [22] as HLH. There is an ongoing debate about which hepatic parameter is most critical for NKTCL with HLH prognosis. Some studies suggest elevated ALT or bilirubin is not a prognostic factor for HLH [1, 7]. In contrast, others indicate that DBIL and AST are associated with poor outcomes in adult HLH patients [8, 9]. We examined and verified that hepatic parameters were highly informative in distinguishing NK/T‐HLH from NK/T‐NHLH. Hepatic involvement with GGT ≥ 183 U/L, AST ≥ 92 U/L, and DBIL ≥ 6.4 μmol/L exhibited sufficient discriminatory power for predicting mortality in NKTCL patients. Then, we provided a simplified tool by combining GGT, AST, and DBIL, termed the GSD index, which could be a more effective predictor of worse survival in NKTCL patients.

Multiple studies show that serum ferritin, platelets, ALT, lymphocyte ratio, and creatinine can be independent prognostic factors for HLH [10, 23]. Most of this research mainly focused on primary HLH prognosis. One study identified high EBV load as a risk factor for lymphoma‐associated HLH [9]. At the same time, another found that IL‐2R (> 3900 U/mL) and ferritin (> 1000 ng/mL) elevation [24], which was termed the optimized HLH inflammatory (OHI) index, was highly predictive of mortality in hematologic malignancies related to HLH. However, our study found no significant EBV difference in NKTCL patients with or without HLH. Furthermore, lymphoma patients often have high IL‐2R [24], and ferritin levels can rise due to hematologic malignancies, blood transfusions, or other conditions [25]. Additionally, markers such as IL‐2R and EBV‐DNA may not be available in all hospitals. Therefore, an easily measurable indicator that is rarely affected by other factors needs to be developed. The GSD index parameters (GSD, AST, DBIL) can be rapidly and routinely obtained in any hospital. Compared with established HLH prognostic indicators—including CNS involvement, BM infiltration, high‐risk stratification, and others—our result showed that the GSD index demonstrated superior predictive capacity for mortality and can be an independent prognostic factor. Correspondingly, significantly reduced median OS was seen in GSD‐positive patients compared to non‐fulfillers.

When activated, hemophagocytic histiocytes cause a cytokine storm in HLH; they can damage many organs [16]. IFN‐γ, IL‐6, and TNF are vital cytokines that are often elevated in cytokine storms and are thought to have central immunopathologic roles. IL‐10 inhibits the production of TNF and IL‐6 and down‐regulates antigen presentation [15]. IFN‐γ is a cytokine primarily produced by immune cells, including innate lymphocytes, such as NK cells, innate lymphoid cells, and adaptive immune cells [26].

The importance of IFN‐γ in the pathogenesis of HLH has been initially strengthened by data obtained in experimental mouse models [27]. Research shows that IFN‐γ may contribute to liver impairment in HLH [28]. In our current study, correlative analysis showed that IFN‐γ was closely related to the concentration of GGT, AST, and DBIL; similar results have been demonstrated in pediatric patients with HLH [28]. IL‐10 exhibited no correlation with liver parameters, suggesting its role in hepatic dysfunction may be more complex in this context. These findings underscore the intricate interplay between specific cytokines and hepatic dysfunction in the context of NKTCL. Therefore, therapy targeting IFN‐γ might be a treatment for NK/T‐HLH patients with hepatic impairment.

The HLH‐94 [29] and the HLH‐2004 regimen [30] are still the current first‐line treatments for HLH, which mainly rely on etoposide and dexamethasone, giving an objective response rate (ORR) of 45.9% for HLH among patients with NKTCL [12]. Wang et al. showed that the DEP regimen [13] could improve ORR when used as first‐line therapy for lymphoma‐associated HLH compared to the HLH‐1994 regimen. Rong Tao et al. [31] reported a series of patients with NK/T‐HLH treated uniformly by MEDA chemotherapy who showed good early response and survival. However, the hepatotoxicity of etoposide complicates the treatment of patients with HLH‐induced ALF. The combination of glucocorticoids, etoposide, fludarabine, and gamma‐globin did not work well for HLH patients with liver failure, with only 45.45% survival in a retrospective study [14]. In our study, after HLH onset, 53% of patients received therapeutics according to the HLH‐94 protocol. Only 23.5% of the patients were eligible for combined chemotherapy. The OS was still poor, with a median survival of 11 months. Therefore, innovative treatments are urgently needed to improve the prognosis of NK/T‐HLH patients with hepatic involvement. Emapalumab, a human anti‐IFNγ antibody, has been approved as a cytokine‐targeting agent for HLH [32] and has demonstrated a survival rate of over 70% for patients with primary HLH [33]. Although research suggests IFN‐γ may contribute to hepatic impairment in HLH [28], no studies have demonstrated that anti‐IFN‐γ treatment rapidly reverses such impairment. One report described successful treatment with emapalumab in a case of refractory EBV‐associated HLH presenting with multiorgan failure; hepatic dysfunction resolved within a month [34]. More recently, a 2024 brief report highlighted emapalumab as salvage therapy for adults with malignancy‐associated HLH. Critically, it documented a rapid decline in ALT, AST, and TBIL following treatment, mirroring our observations [35]. In our research, we used emapalumab for two patients with NK/T‐HLH who experienced severe hepatic injury. Drug infusions were well tolerated, and no significant adverse event occurred. The liver function of the two patients improved rapidly after the administration of emapalumab. This is the first report that successfully shows that emapalumab is a safe and effective way of salvaging therapy for hepatic impairment caused by NK/T‐HLH. It suggests combining IFN‐γ antibodies is a potential therapeutic strategy for NK/T‐HLH.

This study aims to provide a prognostic index of NK/T‐HLH based on liver function and treatment strategies for complicated HLH; suppressing the cytokine storm is beneficial in treating hepatic impairment caused by NK/T‐HLH. It is noted that a small sample size limits the current study, and the risks/benefits of intensifying or altering therapy based on the GSD index are still unknown and warrant further larger, multicenter cohort study.

## Author Contributions

**Yehua Yu:** conceptualization, writing – original draft. **Liyuan Ma:** writing – review and editing. **Haifang Hang:** investigation. **Yuyang Pang:** investigation. **Wei Lu:** investigation. **Jiajia Liu:** formal analysis. **Hui Zhou:** investigation. **Jun Shi:** conceptualization, funding acquisition, writing – original draft.

## Ethics Statement

This is a retrospective study analyzing de‐identified clinical data from existing medical records. No human tissue samples were collected, and no additional patient interventions or experiments were conducted. The data were anonymized prior to analysis to ensure patient confidentiality. This study was approved by the Independent Ethical Committee of Shanghai Ninth People's Hospital (SH9H‐2024‐T63‐1), and the ethics committee granted a waiver of informed consent.

## Conflicts of Interest

The authors declare no conflicts of interest.

## Supporting information


**Data S1:** cam471606‐sup‐0001‐DataS1.docx.

## Data Availability

Data presented in this study are included in the article itself and/or Supporting Information. Further data should be accessed via email to the authors.
