# Supplementary material for: IFN‐γ Driven Hepatic Injury Exacerbates Mortality in NK/T‐Cell Lymphoma‐Associated Hemophagocytic Lymphohistiocytosis
Source: Cancer Med. 2026 Feb 15;15(2):e71606. doi: 10.1002/cam4.71606 (PMC12906978; doi:10.1002/cam4.71606)
Supplement: Supplementary file 1 — Data S1: cam471606‐sup‐0001‐DataS1.docx. [file CAM4-15-e71606-s001.docx]

IFN-γ Driven Hepatic Injury Exacerbates Mortality in NK/T-Cell Lymphoma-associated Hemophagocytic Lymphohistiocytosis

Yehua Yu, Liyuan Ma, Haifang Hang, Yuyang Pang, Wei Lu, Jiajia Liu, Hui Zhou, Jun Shi


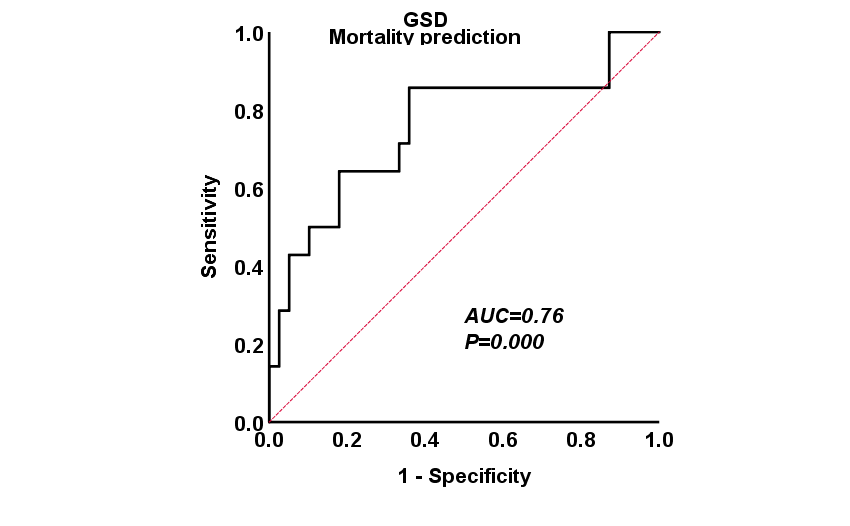


Supplemental Figure 1. ROC analysis of the bootstrap-resampled GSD index for predicting mortality in NKTCL patients

Supplemental Table 1. Sensitivity analysis of bootstrap-resampled GSD index via logistic regression

|  | **Number of Samples** | **Std. Error** | **Sig. (2-tailed)** | **95% CI** | |
| --- | --- | --- | --- | --- | --- |
|  |  |  |  | **Lower** | **Upper** |
| **GSD** | 1000 | .325 | .002 | -1.727 | -.421 |
|  | 2000 | .326 | .001 | -1.727 | -.501 |
|  | 5000 | .317 | .001 | -1.727 | -.421 |
|  | 10000 | .322 | .001 | -1.727 | -.421 |

Supplemental Table 2. Characteristics between NKTCL patients who completed follow-up and those lost to follow-up

|  | **Complete follow-up (n=39)** | **Loss to follow-up(n=14)** | ***P*** |
| --- | --- | --- | --- |
| **Gender(M/F)** | 28/11 | 8/6 | 0.34 |
| **Age(years), Median(range)** | 54(28-79) | 53(19-79) | 0.89 |
| **HLH, n(%)** | 14(77.8) | 4(22.2) | 0.75 |
| **Prognosis Group, n (%)** |  |  |  |
| Low-Risk | 8 (20.5) | 1(7.1) | 0.61 |
| Intermediate-Risk | 8 (20.5） | 3(21.4) |  |
| High-Risk | 23(59.0) | 10(71.4) |  |
| **Ann Arbor stage, n (%**) |  |  |  |
| I-II | 20 (51.3) | 8(57.1) | 0.76 |
| III-IV | 19(48.7) | 6(42.9) |  |

Supplemental Table 3. Characteristics between NKTCL patients who with and without cytokine data

|  | **with cytokine data   (n=46)** | **without cytokine data  (n=7)** | ***P*** | |
| --- | --- | --- | --- | --- |
| **Gender(M/F)** | 30/16 | 6/1 | 0.41 | |
| **Age(years), Median(range)** | 52(19-79) | 57(42-79) | 0.17 | |
| **HLH,n(%)** | 18(39.1) | 0(0) | 0.08 | |
| **Prognosis Group, n (%)** |  |  |  | |
| Low-Risk | 6（13.1） | 3（42.9） | 0.16 | |
| Intermediate-Risk | 10（21.7） | 1（14.2） |  | |
| High-Risk | 30（65.2） | 3（42.9） |  | |
| **Ann Arbor stage, n (%**) |  |  |  | |
| I-II | 24（52.2） | 4（57.1） | >0.99 | |
| III-IV | 22（47.8） | 3（42.9） |  | |
| **GGT(U/L), Median(range)** | 44(6-2178) | 37(23-253) | 0.93 | |
| **AST(U/L), Median(range)** | 43(14-1164) | 37(16-52) | 0.10 |  |
| **DBIL(㎛ol/L), Median(range)** | 3.75(0.5-86.8) | 3.2(2-6) | 0.72 | |

Supplemental table 4 Treatment and response of HLH in NK/TCL patients

| **ID** | **GSD**  **index+** | **Gender/Age** | **Nasal** | **Stage** | **Risk** | **Treatment for HLH** | **Response of**  **HLH to therapy** | **Dead/Alive** | **OS(Months)** |
| --- | --- | --- | --- | --- | --- | --- | --- | --- | --- |
| 1 | Yes | Female/41 | Yes | IV B | High | DED, Ruxolitinib, Emapalumab | CR | alive | 8 |
| 2 | Yes | Male/37 | Yes | IV B | High | DED, Emapalumab | PR | dead | 9 |
| 3 | Yes | Male/60 | No | IV B | High | VP-16+DX | CR | alive | 53 |
| 4 | No | Female/61 | No | IV B | High | VP-16+DX, DEP | PR | alive | 7 |
| 5 | No | Male/41 | No | IV B | High | DEP, Ruxolitinib | PR | dead | 21 |
| 6 | No | Female/49 | No | IV B | High | VP-16+DX | NR | alive | 9 |
| 7 | No | Male/60 | Yes | II B | High | GeMoxD | CR | alive | 17 |
| 8 | No | Male/29 | No | II A | High | GeMoxD-Peg | CR | alive | 15 |
| 9 | No | Female/47 | Yes | IV B | High | VP-16+DX | PR | alive | 2 |
| 10 | Yes | Male/75 | No | IV B | High | None | NR | dead | 2 |
| 11 | Yes | Male/79 | Yes | IV B | High | CEOP | NR | dead | 24 |
| 12 | No | Female/67 | Yes | IV B | High | GVD | NR | dead | 8 |
| 13 | No | Male/43 | No | IV B | High | VP-16+DX | NR | dead | 13 |
| 14 | No | Male/53 | No | IV A | High | VP-16+DX | NR | dead | 11 |
| 15 | Yes | Female/57 | Yes | II A | Intermediate | VP-16+DX | NR | dead | 11 |
| 16 | No | Male/37 | Yes | IVB | High | VP-16+DX | CR | alive | 10 |
| 17 | Yes | Male/71 | Yes | IVB | High | VP-16+DX | NR | dead | 5 |
| 18 | Yes | Male/44 | NO | IVA | Low | CMOP+VP-16+Emapalumab | PR | dead | 7 |

GeMoxD: Gemcitabine +etoposide+ Oxaliplatin+ dexamethasone; Peg: L-asparaginase; DED/P: liposomal daunorubicin+ etoposide+ dexamethasone/ prednisone, VP-16+DX: etoposide+ dexamethasone; GVD: Gemcitabine + vincristine+ dexamethasone; CEOP: cyclophosphamide +etoposide + vincristine + prednisone.
